# Straightforward Synthesis of Mn_3_O_4_/ZnO/Eu_2_O_3_-Based Ternary Heterostructure Nano-Photocatalyst and Its Application for the Photodegradation of Methyl Orange and Methylene Blue Dyes

**DOI:** 10.3390/molecules26154661

**Published:** 2021-07-31

**Authors:** Jayachamarajapura Pranesh Shubha, Haralahalli Shivappa Savitha, Syed Farooq Adil, Mujeeb Khan, Mohammad Rafe Hatshan, Kiran Kavalli, Baji Shaik

**Affiliations:** 1 Department of Chemistry, Don Bosco Institute of Technology, Mysore Road, Bangalore 560074, India; 2 Department of Chemistry, SJB Institute of Technology, Kengeri, Bangalore 560060, India; hssavitha@sjbit.edu.in; 3 Department of Chemistry, College of Science, King Saud University, P.O. Box 2455, Riyadh 11451, Saudi Arabia; kmujeeb@ksu.edu.sa (M.K.); mhatshan@ksu.edu.sa (M.R.H.); 4 Department of Mechanical and Automobile Engineering, School of Engineering and Technology, CHRIST University, Bangalore 560074, India; kiran.k@christuniversity.in; 5 Department of Advanced Materials Engineering for Information and Electronics, Kyung Hee University, 1732 Deogyeong-daero, Giheung-gu, Yongin-si 446-701, Gyeonggi-do, Korea; shaikbaji2@khu.ac.kr

**Keywords:** ternary heterostructure, Mn_3_O_4_/ZnO/Eu_2_O_3_, photocatalyst, methylene blue, methyl orange, dye degradation

## Abstract

Zinc oxide-ternary heterostructure Mn_3_O_4_/ZnO/Eu_2_O_3_ nanocomposites were successfully prepared via waste curd as fuel by a facile one-pot combustion procedure. The fabricated heterostructures were characterized utilizing XRD, UV–Visible, FT-IR, FE-SEM, HRTEM and EDX analysis. The photocatalytic degradation efficacy of the synthesized ternary nanocomposite was evaluated utilizing model organic pollutants of methylene blue (MB) and methyl orange (MO) in water as examples of cationic dyes and anionic dyes, respectively, under natural solar irradiation. The effect of various experimental factors, viz. the effect of a light source, catalyst dosage, irradiation time, pH of dye solution and dye concentration on the photodegradation activity, was systematically studied. The ternary Mn_3_O_4_/ZnO/Eu_2_O_3_ photocatalyst exhibited excellent MB and MO degradation activity of 98% and 96%, respectively, at 150 min under natural sunlight irradiation. Experiments further conclude that the fabricated nanocomposite exhibits pH-dependent photocatalytic efficacy, and for best results, concentrations of dye and catalysts have to be maintained in a specific range. The prepared photocatalysts are exemplary and could be employed for wastewater handling and several ecological applications.

## 1. Introduction

Pollution is the result of various industrial advancement activities of man. Among the various types of pollutions, water pollution is one of the major concerns as its effects are widely felt. Among the various pollutants such as heavy metals, nanoparticles, etc., synthetic dyes, particularly organic compound-based dyes used in the textile industry, have a staggering impact on aquatic life as well as human beings. These are persistent and have the capacity to absorb dissolved O_2_ from water bodies, which has a considerable impact on aquatic life. These organic pollutants survive for a longer interval and possibly become xenobiotic and may also lead to biomagnification [1,2]. Therefore, an untreated fabric effluent is extremely dangerous to both earthly and aquatic organisms by negatively influencing the naturalistic ecological system, which causes long-term health issues [3].

Various biological, chemical and physical experiments have been conducted for photodecomposition of synthetic and organic dyes [4,5,6,7,8,9,10,11]. Recently, advanced oxidation methods have been the talk of the scientific community searching for cost-effective and efficient degradation of dyes present in effluents. The metal oxide-based semiconductor photocatalysts for decomposition of noxious dye molecules have drawn the interest of researches as a potential process to resolve a tremendously increasing issue of water contamination due to deleterious organic dyes. Moreover, due to the photosensitive nature of the materials with semiconductor properties, the oxidative degradation of the polluting dye can be performed in sunlight or UV light. 

A variety of semiconductor-based photocatalysts, such as V_2_O_5_, TiO_2_, Fe_2_O_3_, ZnO, CdS, etc., were notably employed for such photocatalytic removal of dye molecules [12]. Among these, zinc oxide (ZnO) has displayed prominent environmental remediation and hence draws a lot of attention in the field of photocatalytic degradation [13]. ZnO possesses several merits such as inexpensive, superb electronic structure, non-toxic, high chemical stability, exemplary photo-sensitivity, biocompatibility and thermal stability. These properties make ZnO an exemplary material for a photocatalyst. There are pros and cons to using ZnO [14,15,16]. There is a large band gap and the photo-generated charge carriers reunite at a faster rate and thus limit the use of ZnO. To improve the charge separation and expand the response of ZnO towards visible light, the ZnO is doped with both metals and non-metals [17,18,19,20,21,22,23]. It leads to the formation of heterojunctions with the second semiconductor component. The formation of heterojunctions will provide opportunities to increase the separation efficacy of photo-induced charge carriers. In recent years, various ZnO-binary and ternary heterojunctions were synthesized including, binary ZnO/ZnS heterostructures, ternary ZnO–ZnS–Gd_2_S_3_ heterostructures and ternary ZnO/Cu_2_O/Si nanowire arrays [24,25,26,27,28,29].

Reasonably prepared ZnO-ternary heterojunctions offer considerable potential by assisting the movement of electrons attributed to the existence of multicomponent photo-systems and, therefore, are helpful in photocatalytic decomposition of deleterious dye molecules. The heterojunction-based ZnO photosystems efficiently expand the lifespan of photo-induced charge carriers and enhance the absorption of light [30,31,32]. Controlled synthesis of ternary heterojunctions is the need of the hour. These kinds of systems were synthesized by several chemical and physical procedures such as CVD, sol-gel, microwave-assisted heating, coprecipitation, solvothermal and hydrothermal procedures. It is noteworthy that these methods require sophisticated technology tools, a longer duration of reaction and high-temperature conditions. Contrary to the above-mentioned methods, the solution combustion procedure is cost-effective, less time-consuming, consumes less energy and requires less effort to carry out the process. It is also easy to scale up the process. Eco-friendly materials can be utilized as starting materials and can be efficiently used in this process. These parameters, which are highly useful, persuaded exploring the fabrication of ZnO ternary heterojunction utilizing other photo-active metal oxides and lanthanides [33,34,35].

**Scheme 1:**
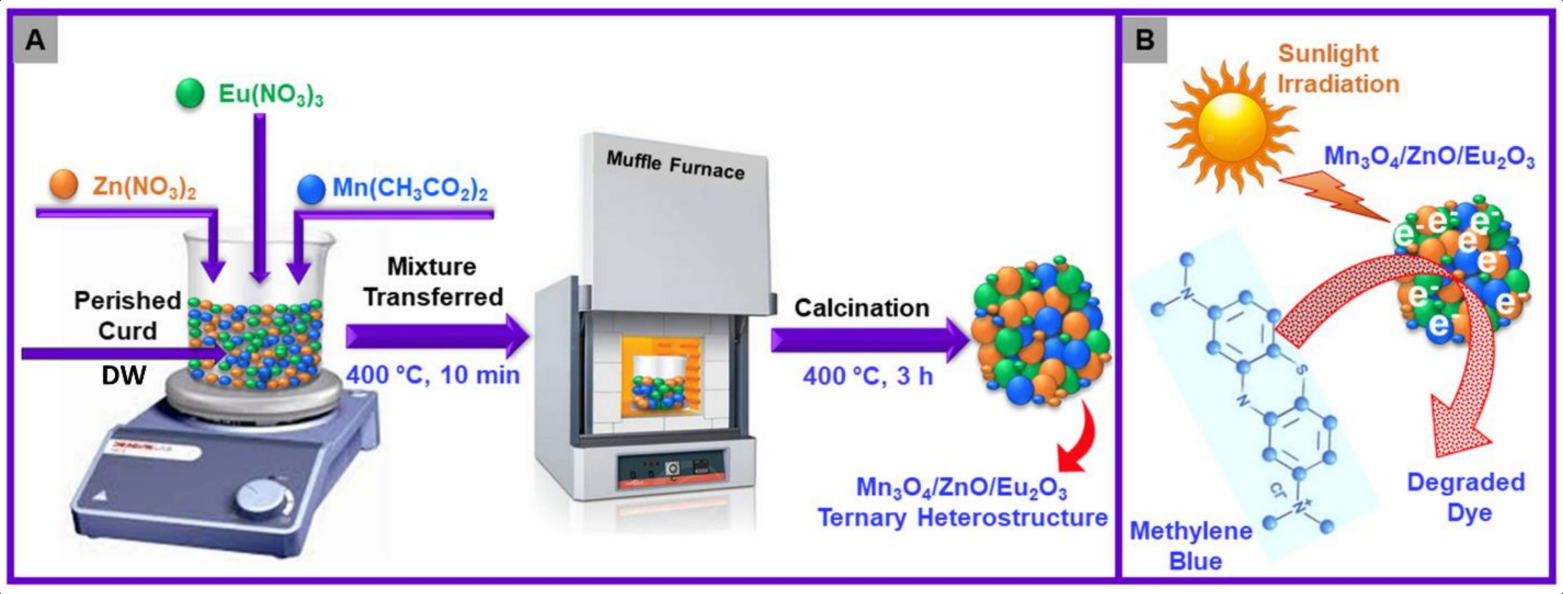
(**A**) Schematic diagram of fabrication of Mn_3_O_4_/ZnO/Eu_2_O_3_ nanocomposites. (**B**) Scheme presenting the photodegradation of MB dye.

Herein, an attempt was made to prepare an environmentally friendly and cheap ternary Mn_3_O_4_/ZnO/Eu_2_O_3_ nanocomposite via a simple single-step combustion procedure to achieve higher photodegradation activity over two chronic dyes such as MB and MO (Scheme 1). The as-prepared Mn_3_O_4_/ZnO/Eu_2_O_3_ nanocomposites were employed as a photocatalyst for decomposing dyes in an aqueous solution at natural sunlight radiation. The ternary system demonstrated an enhanced photocatalytic performance. The photocatalytic results displayed that the Mn_3_O_4_/ZnO/Eu_2_O_3_ photocatalyst showed higher photocatalytic performance for the decomposing of MB and MO dyes under natural sunlight irradiation than a UV irradiation and dark, and 98% and 96% of MB and MO dyes were decomposed within 150 min, respectively, under the optimized conditions. This work offers new insights in designing multicomponent ZnO-based photocatalysts towards environmental remediation.

## 2. Results and Discussion

### 2.1. Photocatalyst Characterization

**Figure 1:**
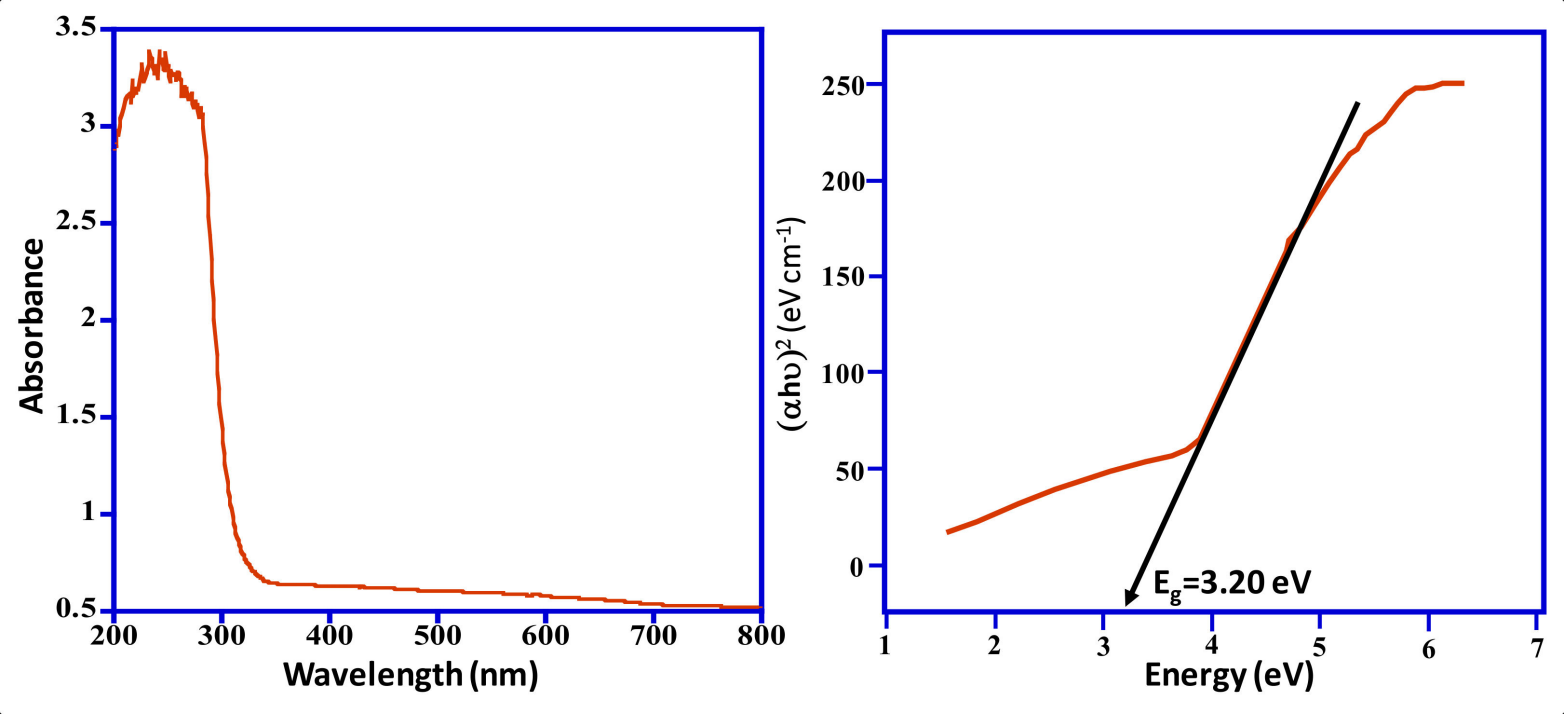
UV–Vis absorption and bandgap spectrum of the fabricated Mn_3_O_4_/ZnO/Eu_2_O_3_ photocatalyst.

Figure 1 illustrates the UV–Vis absorption spectra of synthesized Mn_3_O_4_/ZnO/Eu_2_O_3_ nanocomposite. The synthesized photocatalyst exhibits the absorption range from 800 nm to 200 nm with maximal absorption at ~242 nm along with an absorption edge between 350 nm to 370 nm; this indicates that the synthesized material is photolytically active in the UV region and visible region; furthermore, it also illustrates the crystalline nature of the synthesized oxides [36]. The bandgap of the sample is about 3.20 eV, which is calculated from the Kubelka–Munk equation. A wide range of absorption of the light assists in efficient photocatalytic decomposition and improves performance. A comparative UV–Vis spectra of ZnO nanoparticles is given in Figure S1. 

**Figure 2:**
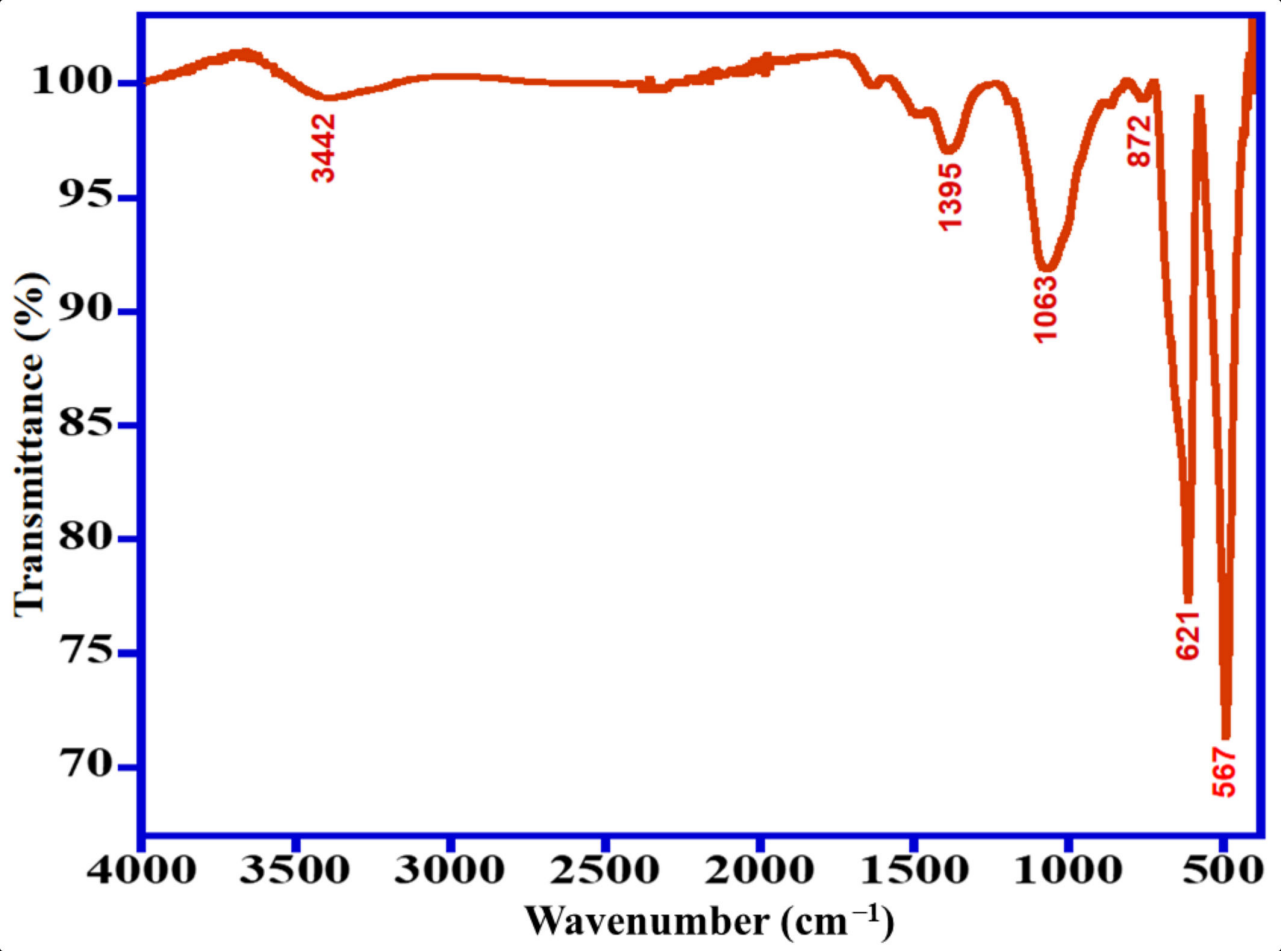
FT-IR spectra of the synthesized Mn_3_O_4_/ZnO/Eu_2_O_3_ photocatalyst.

The FT-IR spectra were usually employed to identify the functional groups existing on the prepared photocatalyst surface. The FT-IR spectra of Mn_3_O_4_/ZnO/Eu_2_O_3_ were illustrated in Figure 2. The absorption band located at approximately 3442 cm^−1^ could be owing to (O–H) groups stretching vibrations of adsorbed H_2_O molecules, and the absorption peak at 1395 cm^−1^ belongs to (C–OH) stretching vibrations. Additionally, an absorption peak at nearly 1063 cm^−1^ is assigned to (C=O) stretching vibrations of acetate. The fingerprint peaks situated at 567 cm^−1^ and 621 cm^−1^ corresponding to (Zn–O) and (Zn–O–Zn) stretching vibrations, respectively. Finally, a peak situated at 872 cm^−1^ is associated with the stretching vibrations of (Zn–O).

**Figure 3:**
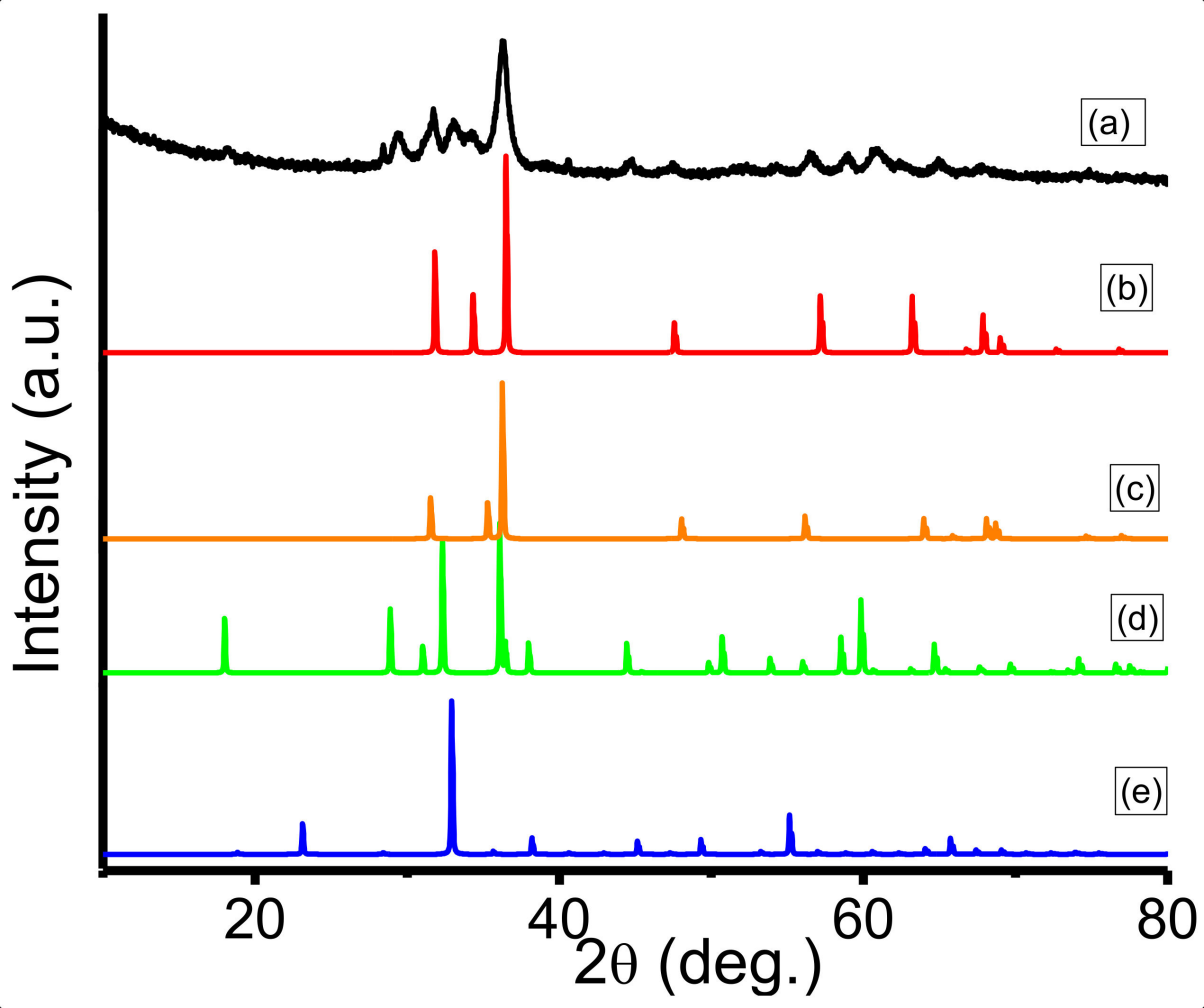
XRD analysis of the synthesized Mn_3_O_4_/ZnO/Eu_2_O_3_ photocatalyst (**a**) and a comparative ZnO (**b**), Eu_2_O_3_ (**c**), Mn_3_O_4_ (**d**), Mn_2_O_3_ (**e**) diffraction patterns.

Figure 3 demonstrates the XRD patterns of Mn_3_O_4_, ZnO, Eu_2_O_3_ and Mn_3_O_4_/ZnO/Eu_2_O_3_ NPs. All diffraction reflections of Figure 3a are crystalline in nature and a mixture from hexagonal and tetragonal structures of ZnO, Mn_3_O_4_ and Eu_2_O_3_. Figure 3b displayed that all the fingerprint peaks could be accredited to the ZnO-hexagonal phase with space group of P63mc as well as lattice parameters a = 3.249 Å and c = 5.205 Å, which is very much in accordance with the recorded data (JCPDS file number 5–664). Besides, Figure 3c,d illustrates the XRD pattern of Mn_3_O_4_ and Eu_2_O_3,_ which are in tetragonal and hexagonal structures with (JCPDS file number 8–17) with lattice parameters a = 5.76 Å and c = 9.44 Å having the space group I41amd (no. 141) and (JCPDS file number 65–369) with lattice parameter a = 3.27300 Å and c = 5.08300 having the space group P63/mmc (no. 194), respectively.

**Figure 4:**
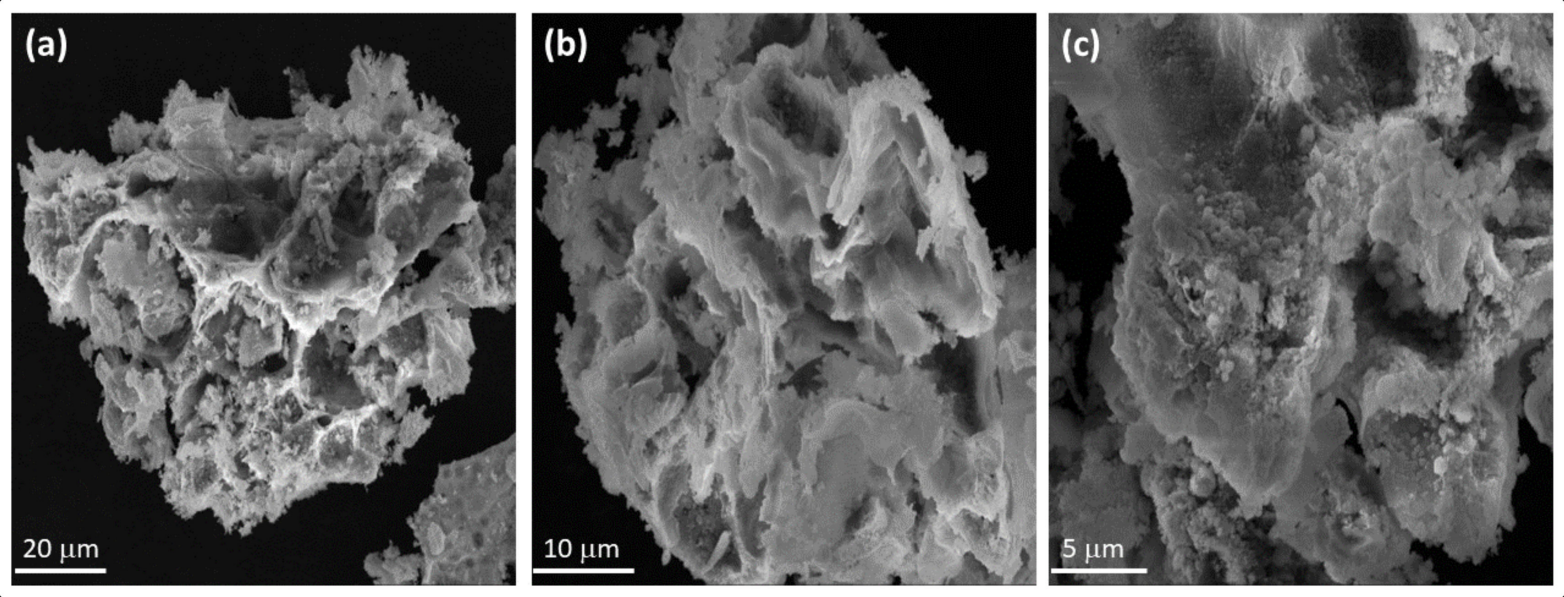
FE-SEM micrographs of the synthesized Mn_3_O_4_/ZnO/Eu_2_O_3_ nanocomposite. (**a**) Magnification ×2800 (**b**) Magnification ×6000 and (**c**) Magnification ×12,000.

Morphological properties of the synthesized Mn_3_O_4_/ZnO/Eu_2_O_3_ photocatalyst are studied by FE-SEM analysis, and the results were illustrated in Figure 4a–c. Low magnification FE-SEM micrograph as displayed in Figure 4a,b reveals that the Mn_3_O_4_/ZnO/Eu_2_O_3_ is composed of clusters of flakes, in which all the flakes are interconnected and form a net-like structure with large pores (Figure 4c). According to the formerly reported studies, it can be said that the flakes could belong to the Mn_3_O_4_ NPs of the material, whilst the clusters could be the Eu_2_O_3_ and ZnO NPs in the synthesized nanocomposite [37].

**Figure 5:**
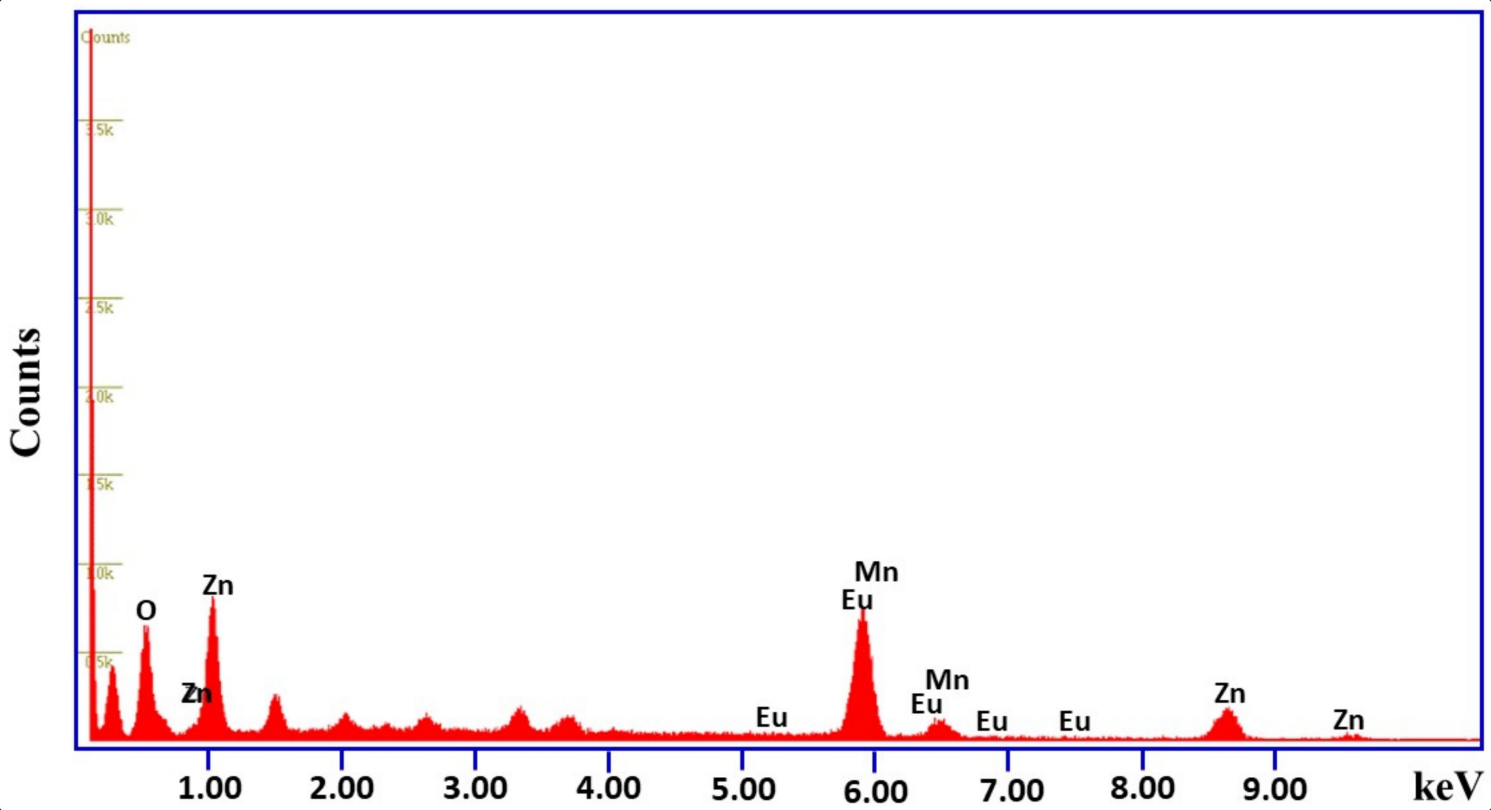
EDX spectrum of the synthesized Mn_3_O_4_/ZnO/Eu_2_O_3_ nanocomposite.

The elemental analyses of the fabricated Mn_3_O_4_/ZnO/Eu_2_O_3_ nanocomposite were scrutinized utilizing EDX analysis, as demonstrated in Figure 5, which displays that the synthesized material contains the expected elements, and are well-dispersed throughout the composition, which could be playing a synergetic role in enhancing the photocatalytic activity. Additionally, the energy-dispersive spectra of Mn_3_O_4_/ZnO/Eu_2_O_3_ nanocomposite indicate the existence of all the desired elements such as Mn, Eu, Zn and O as well as the percentage of elemental compositions, which is in accordance with the stoichiometric amounts used in the synthesis of the Mn_3_O_4_/ZnO/Eu_2_O_3_ nanocomposite.

**Figure 6:**
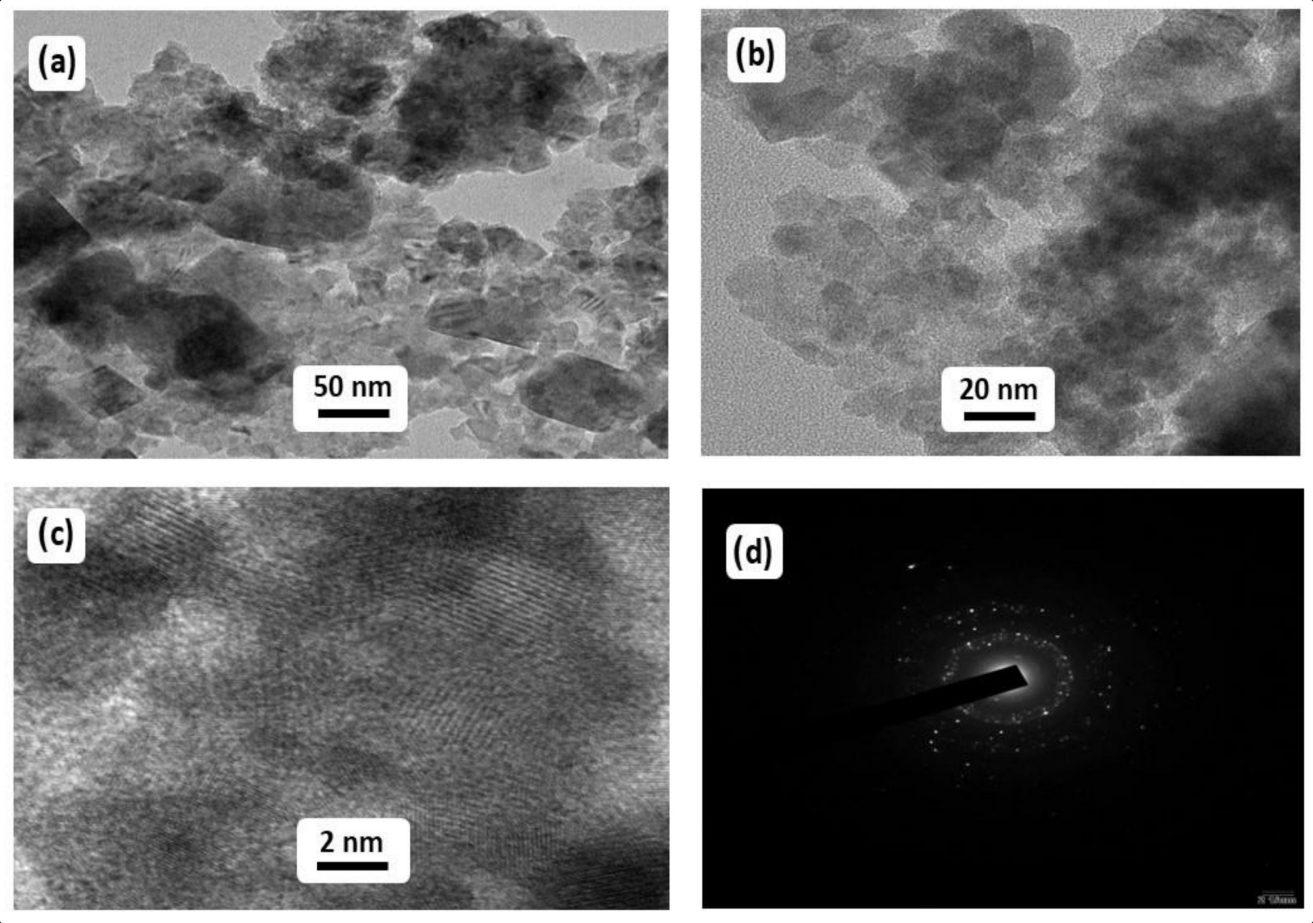
HRTEM images of the prepared Mn_3_O_4_/ZnO/Eu_2_O_3_ nanocomposite. (**a**) 50 nm, (**b**) 20 nm (**c**) 2 nm and (**d**) SAED Pattern.

The HRTEM analysis for the examination of the size and morphology of the prepared Mn_3_O_4_/ZnO/Eu_2_O_3_ nanocomposite at various magnifications is displayed in Figure 6. Figure 6a–c shows the TEM images with varying magnifications, which demonstrate that the spherical morphology particles are uniformly dispersed throughout the material as well as some aggregations can be noticed. The particles sizes are in the range of 20–60 nm. The SAED pattern (Figure 6d) shows the polycrystalline form of the sample, and the reflection planes obtained are in high accordance with the results concluded from the XRD analysis.

### 2.2. Photocatalytic Efficiency Studies of MB and MO Photodegradation Using Mn_3_O_4_/ZnO/Eu_2_O_3_ Heterostructures

The strength of semiconductor photocatalysis strongly depends on the surface area, particle size, bandgap, morphology, crystalline nature and quantity of hydroxyl radical ions on the photocatalyst surface [38]. The formation of electrons and holes on the semiconductor surface by the light absorption and the released holes and electrons will participate in the reaction, or they recombine. If the exterior surface is provided for the charge carriers, they will relocate where the electrons are caught by semiconductors while the holes are trapped by hydroxyl radical ions and form OH^•^ and HO_2_^•^. The ternary heterostructure provided extra surface for the relocation of charge carriers, and therefore, the produced OH^•^ radicals ions are used efficiently to photocatalytic decompose MB and MO dye molecules. 

The hydroxyl radical ions (OH^•^) are non-stable and are a highly active chemical species that have a significant impact on the photocatalytic decomposition of dyes. To recognize whether the OH^•^ radicals are being produced via Mn_3_O_4_/ZnO/Eu_2_O_3_ nanocomposite, coumarin was selected as a compound model, which is a facile and sensitive process for detection of OH^•^. In the existence of OH^•^ radicals produced via the Mn_3_O_4_/ZnO/Eu_2_O_3_ photocatalyst, coumarin transforms to 7-hydroxycoumarin, a luminous substance that shows a photoluminescent peak at a wavelength of 455 nm. In the present work, 0.1 g of the Mn_3_O_4_/ZnO/Eu_2_O_3_ catalyst was added to the coumarin solution (50 mL–0.001 M) irradiated by sunlight.

**Figure 7:**
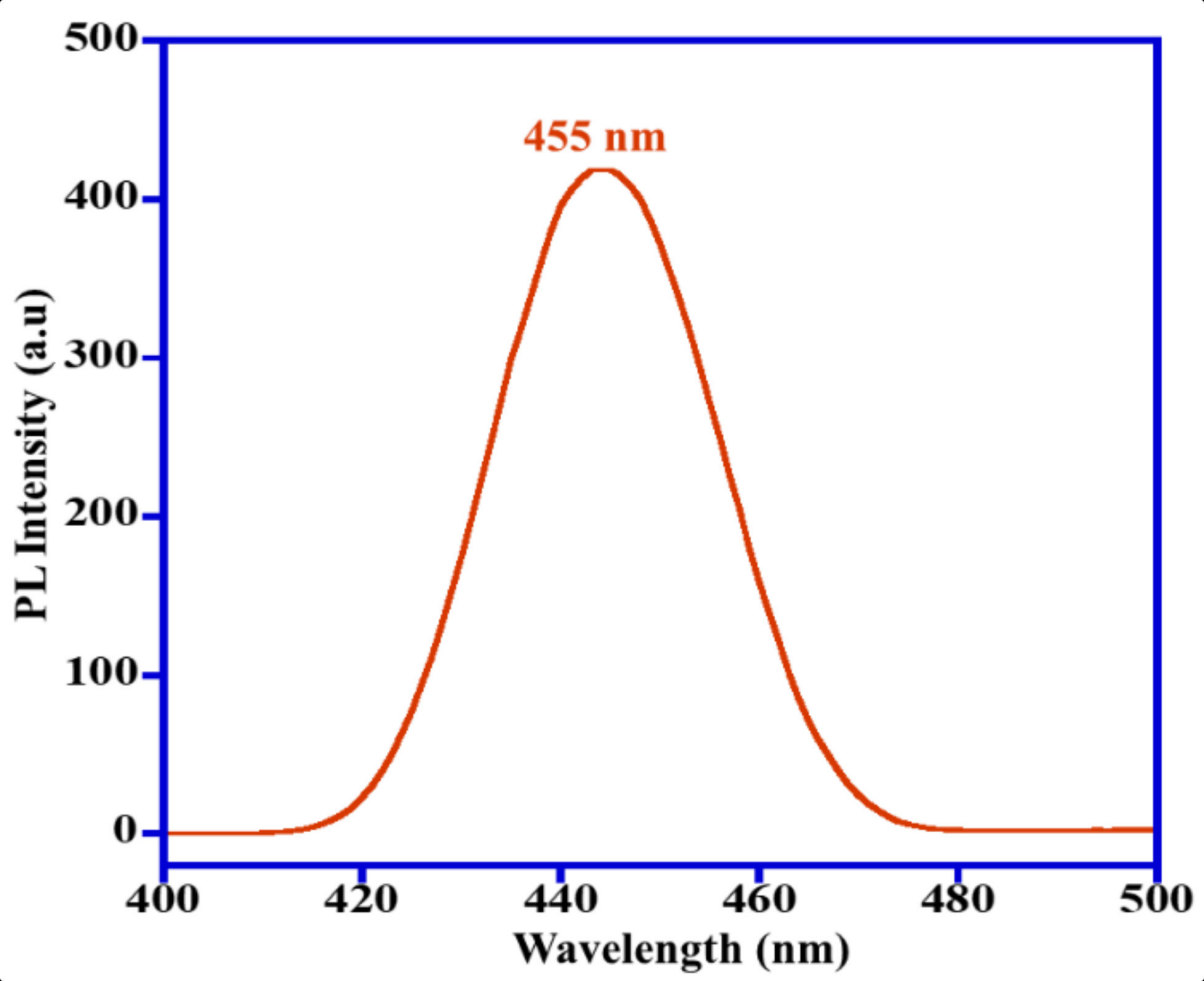
Photoluminescent spectra indicating generation of ^•^OH radicals by Mn_3_O_4_/ZnO/Eu_2_O_3_ photocatalyst.

**Scheme 2:**
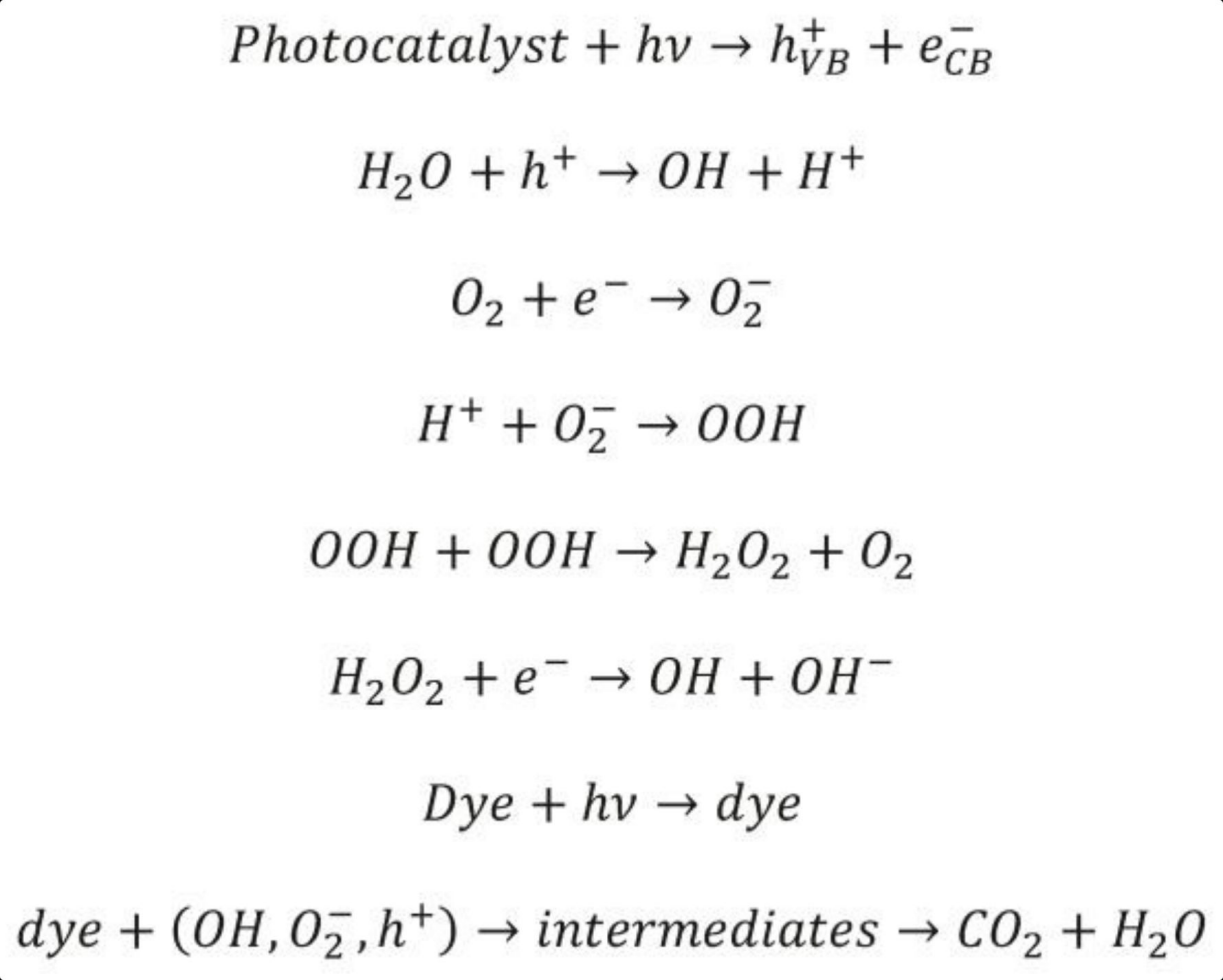
A plausible mechanism of photocatalytic dye degradation.

At a period of 10 min, 2 mL of the sample was injected in a photoluminescence instrument, which displayed the presence of a photoluminescent peak at 455 nm (Figure 7), indicating the generation of OH^•^ radicals, a pivotal chemical species for the decomposition of deleterious dyes [15]. Therefore, it can be concluded that photodegradation of MB and MO dye occurs by a free radical mechanism over Mn_3_O_4_/ZnO/Eu_2_O_3_ photocatalyst. A possible mechanism of photocatalytic degradation is described in Scheme 2.

The attained results from the UV–Vis spectra revealed that the synthesized nanocomposite is active in the UV–Vis range and the visible range. In addition, the calculated bandgap was found to be E_g_ = 3.79 eV. To investigate the photocatalytic efficacy of the synthesized Mn_3_O_4_/ZnO/Eu_2_O_3_ photocatalyst, several reaction variables including dye concentration, light source, catalyst amount and pH value were thoroughly studied, and MB and MO dyes were taken as the standard pollutants for photocatalytic removal in the present work.

In the present work, 100 mL of a solution of various concentrations of dye in the range of (5–20 ppm) are taken for photodegradation reactions. The quantity of fabricated Mn_3_O_4_/ZnO/Eu_2_O_3_ photocatalyst is also altered from 5 to 20 mg. The aqueous solution is mixed with the photocatalyst and varied for 40 min in a dark environment. The kinetics of the photocatalytic removal of dyes has been investigated by periodically taking 3 mL from the solution at times of 30 min; subsequently, the sample was subjected to centrifugation. From the results obtained from UV–Vis spectroscopy, the (C_i_) initial and (C_f_) final dye concentrations are confirmed, and the calculation for the percentage of dye degradation was determined using Equation (1):

(1) $$\%\;\mathrm{Degradation}=\frac{\left(C_{i}-C_{f}\right)}{C_{i}}\times 100$$

#### 2.2.1. Impact of Light Source on Dye Photodegradation

**Figure 8:**
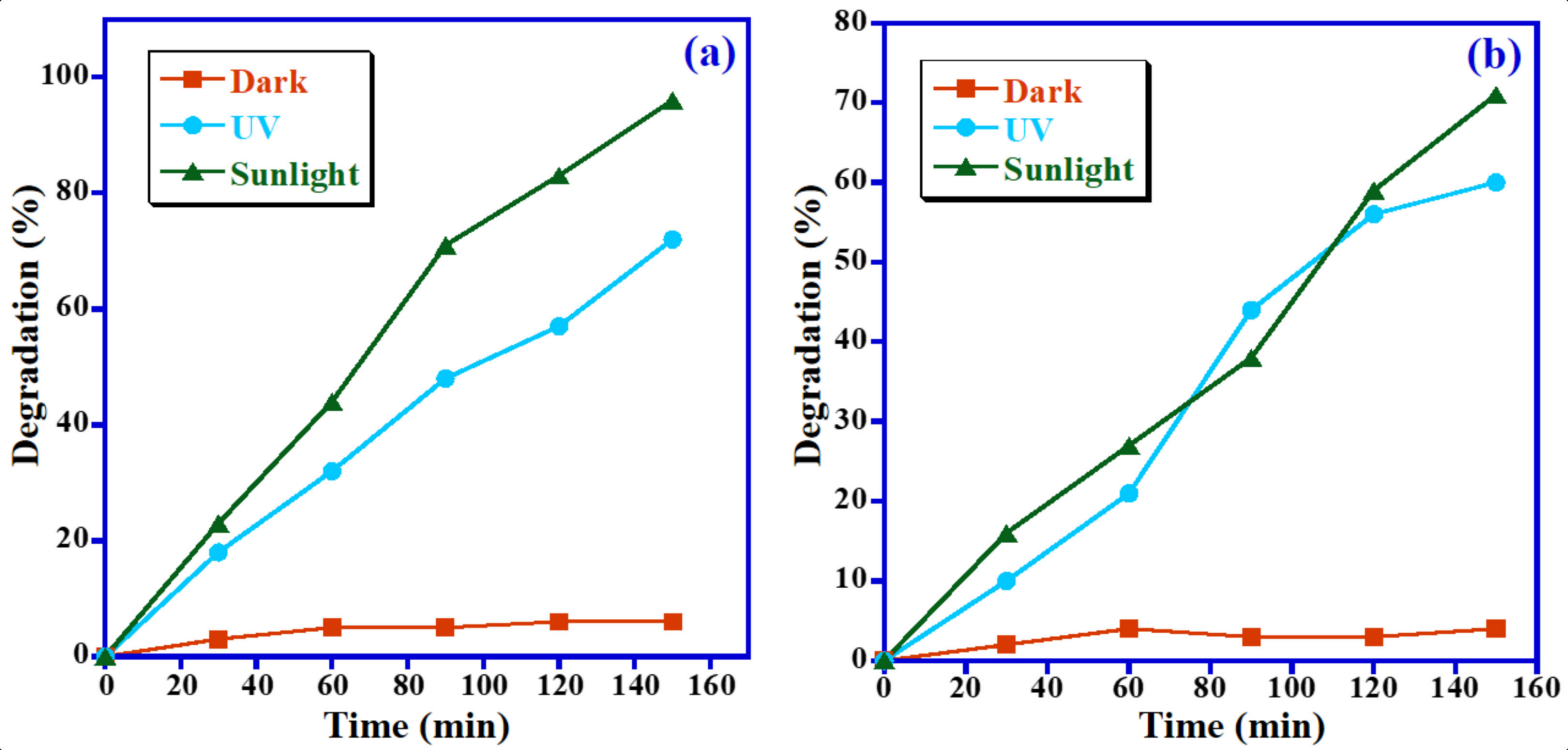
Influence of light source on photodecomposition of (**a**) MB and (**b**) MO.

The synthesized nanocomposite is photocatalytic active in the UV range as well as in the visible range as assured from the UV–Vis spectrum. The initial studies were conducted to emphasize that the light source can exhibit the highest degradation efficacy of the fabricated photocatalyst by keeping identical concentrations of dye 5 ppm, catalyst 15 mg and pH 7 for both MB and MO dyes. Therefore, the photocatalytic removal studies of MB and MO dyes over Mn_3_O_4_/ZnO/Eu_2_O_3_ NPs have been performed in three various conditions, i.e., under natural solar irradiation, UV light and dark. The obtained data illustrated that the synthesized photocatalyst is active in visible light as well as UV irradiation, as confirmed by the obtained UV–Vis spectrum. As expected, when the photocatalytic test was performed in the dark, the photodegradation of MB and MO dyes could be neglected. Additionally, in the case of the photocatalytic experiments conducted in the UV and sunlight irradiation, the attained results reveal that the photodegradation of MB in the sunlight is considerably higher than the decomposition observed in the UV light. In the case of solar irradiation, the synthesized Mn_3_O_4_/ZnO/Eu_2_O_3_ photocatalyst effectively degrades 96% of the MB, which is much more than the degradation obtained under UV irradiation, which yielded a 72% degradation under the identical irradiation time (150 min) as illustrated in Figure 8a, whereas MO dye shows a much lower photodegradation efficiency of 71% within 150 min under natural sunlight irradiation compared to MB dye (Figure 8b). From the aforementioned results, it can be said that the decomposition efficacy of MB and MO under solar light is better than under UV irradiation, which could be due to the presence of both UV and visible lights in a solar irradiation under solar light is better than under UV irradiation, which could be due to the presence of both UV and visible lights in a solar irradiation [39]. As a result, it could be concluded that the Mn_3_O_4_/ZnO/Eu_2_O_3_ photocatalyst is efficient under sunlight irradiation, and further optimization studies are conducted at natural solar irradiation.

#### 2.2.2. Influence of Dye Concentration on Dye Photodegradation

**Figure 9:**
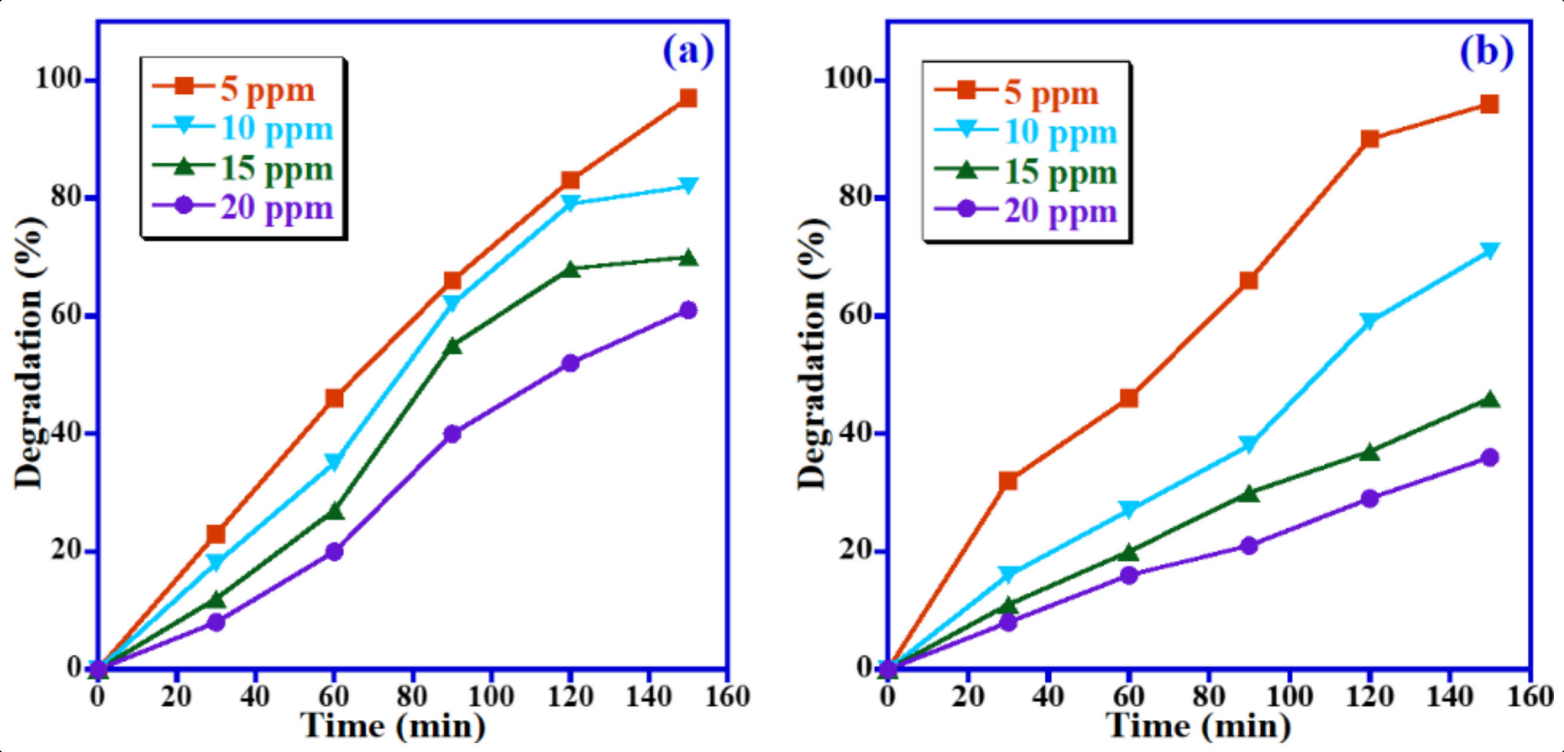
Influence of initial concentration on decomposition of (**a**) MB and (**b**) MO.

The effect of initial concentrations of MB and MO dyes on the photodegradation efficiency of the studied dyes was also investigated by varying the concentration of dye from 5 to 20 ppm under visible light while keeping the photocatalyst dose of 15 mg unchanged at pH 7. The photocatalytic data were graphically illustrated in Figure 9. For MB dye, the photocatalytic data reveal that the removal efficacy of the Mn_3_O_4_/ZnO/Eu_2_O_3_ photocatalyst is inversely proportional to the MB concentration under identical conditions, i.e., the highest degradation was detected at the lowest dye concentration (5 ppm). By raising the MB concentration from 5 to 20 ppm, the photodegradation efficiency of MB declined from 97% to 61%, as displayed in Figure 9a. The same trend occurs in the case of MO dye, in which the photodegradation efficiency of MO reduced from 96% to 36% when the concentration of MO dye raised from 5 to 20 ppm (Figure 9b). This could be owing to the fact that the photocatalytically active sites may be covered with dyes and decrease light absorption on the photocatalyst surface at higher dye concentrations, which leads to reducing the formation of hydroxyl radicals, therefore decreasing degradation efficiency. In contrast, photons readily reach the photocatalyst surface at lower dye concentrations, and the generation of hydroxyl radical ions will be easier [40]. Hence, it is necessary to increase the photocatalyst amount as the concentration of dyes increases.

#### 2.2.3. Impact of Photocatalyst Dose on Dye Degradation

**Figure 10:**
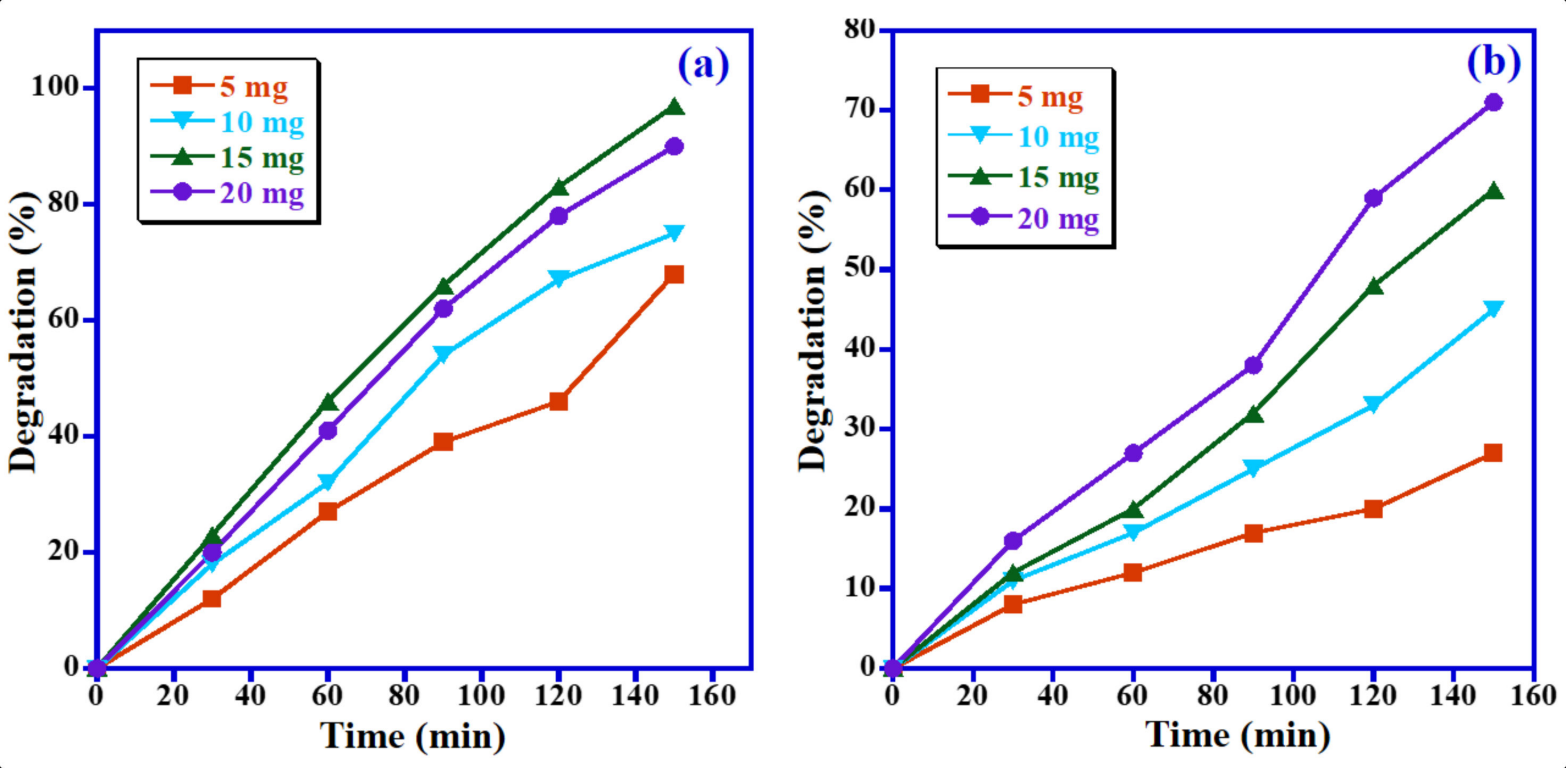
Influence of catalyst quantity on photodecomposition of (**a**) MB and (**b**) MO.

Furthermore, the optimum photocatalyst dose for the photodegradation of MB and MO has also been assessed by altering the catalyst amount from 5 mg to 20 mg at solar light with a constant dye concentration of 5 ppm at pH 7, and the obtained results were graphically presented in Figure 10. Figure 10a displays that the photodegradation of MB dye is strongly affected by the catalyst quantity. We found a 68% decomposition of MB when 5 mg of catalyst was used. However, when the catalyst dosage was raised to 10 mg and 15 mg, 75% and 97% degradation of MB were obtained, respectively. Nevertheless, a further increase in the photocatalyst quantity to 20 mg shows a lower removal efficacy of the catalyst, and a 90% degradation of MB was obtained. For MO, the photodecomposition efficiency of MO was also strongly influenced by the photocatalyst dose. It is distinct that by raising the photocatalyst amount from 5 mg to 20 mg, degradation of MO dye was enhanced from 27% to 71% under identical circumstances (Figure 10b). This was presumably ascribed to the availability of photocatalytic active sites that can produce more radicals by adding more amounts of Mn_3_O_4_/ZnO/Eu_2_O_3_ catalyst; however, further increases of the catalyst dose increases the opacity of the suspension, which in turn leads to the blocking of light penetration [40]. As displayed in Figure 10a,b, MB dye exhibits the maximal photodegradation efficacy at a catalyst quantity of 15 mg, whereas the maximal degradation of MO dye is at 20 mg. However, the difference in catalyst dose is insignificant for MB and MO dyes. Accordingly, a photocatalyst dose of 15 mg has been chosen in the next optimization experiments.

#### 2.2.4. Impact of Solution pH on Dye Degradation

**Figure 11:**
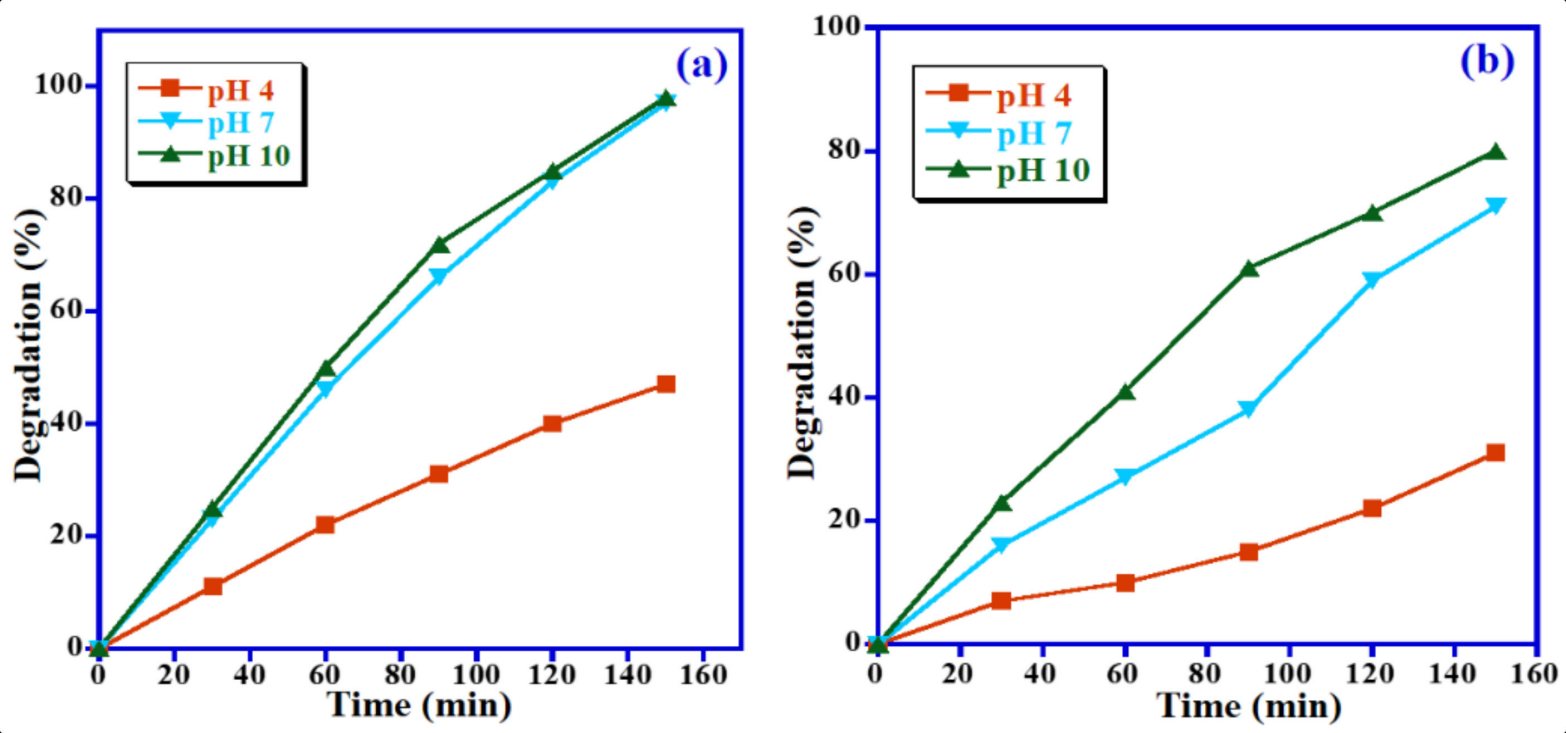
Impact of pH value on the photocatalytic degradation of (**a**) MB and (**b**) MO.

As implied by the previously reported studies, the photocatalytic performance of the photocatalyst is usually related to its ability for the generation of OH^•^ radical ions, which enhances the photocatalytic degradation by many folds in alkaline solutions. Figure 11a,b illustrates the impact of pH value on the decomposition of MB and MO dyes over Mn_3_O_4_/ZnO/Eu_2_O_3_ photocatalyst. The impact of pH value on the removal of MB and MO dyes was also examined at different pH values from 4 to 10 with keeping other factors unchanged (i.e., 15 mg photocatalyst dose, 5 ppm dye concentration and natural sunlight). As expected, the lowest degradation efficiency was obtained at the lowest pH value (i.e., pH 4), with 47% and 31% of the MB and MO being degraded, respectively, at an irradiation time of 150 min. However, when the pH value is increased, the Mn_3_O_4_/ZnO/Eu_2_O_3_ photocatalyst exhibited greater degradation efficacy, and almost 98% decomposition of MB was achieved at pH 7 and pH 10 (Figure 11a). As expected, the highest MO dye degradation efficacy of 80% has been obtained at a higher pH value (pH 10) under identical conditions, as illustrated in Figure 11b. This is probably due to the higher rate of OH^•^ radicals generation and owing to the cumulating of OH^•^ radical ions on the photocatalyst surface at high pH values [41]. Distinctly, the maximum photodegradation of MB (cationic dye) has been achieved in basic media, whereas in the case of MO (anionic dye), it is slightly lower. At pH 10, MB degradation efficiency is 98% upon 150 min irradiation and only 80% for MO under identical circumstances. Accordingly, the pH impact on MB removal is larger than that on MO dye.

**Figure 12:**
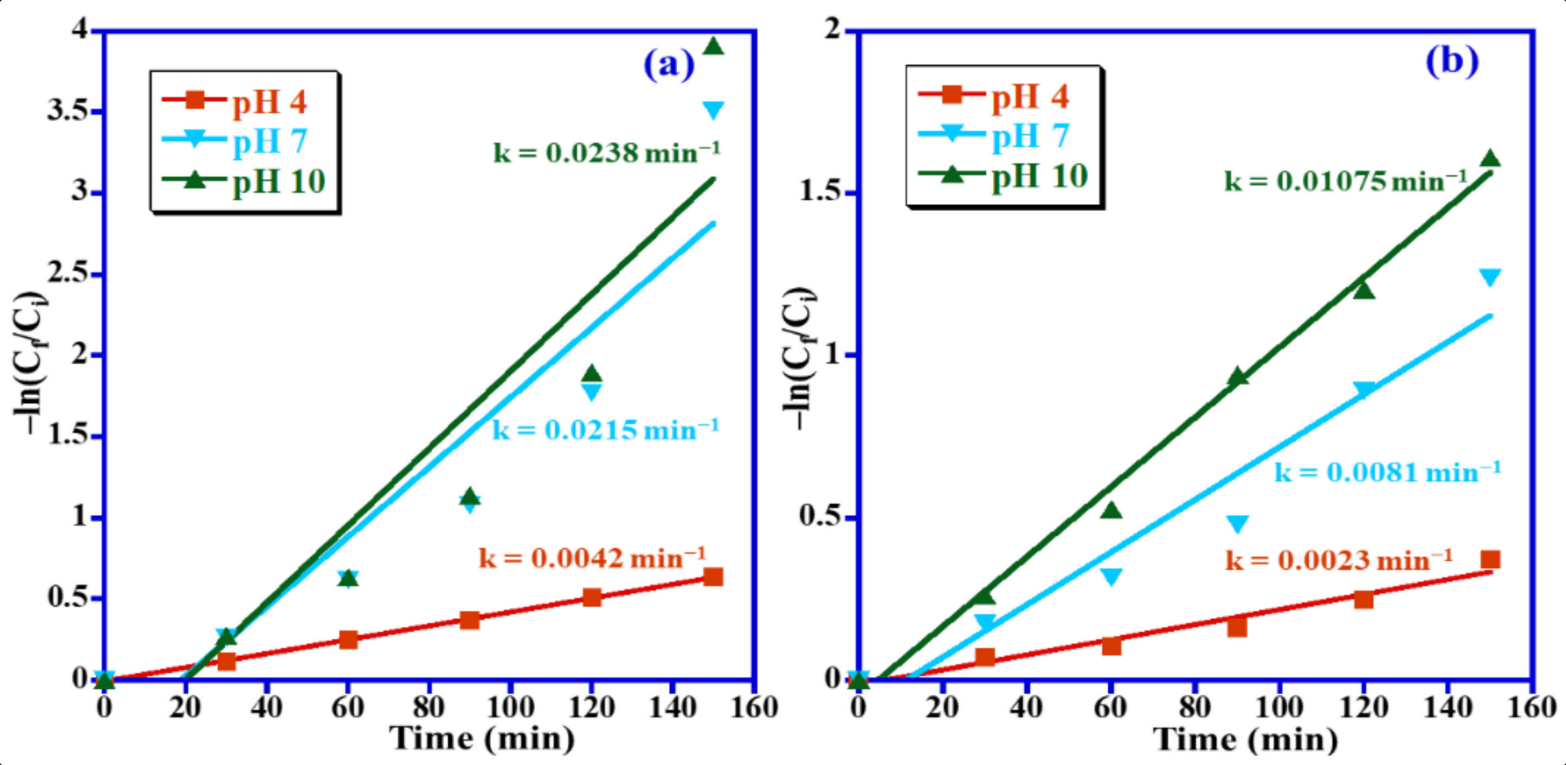
Psuedo first-order kinetics for photocatalytic removal of (**a**) MB and (**b**) MO over Mn_3_O_4_/ZnO/Eu_2_O_3_ photocatalyst.

Kinetic models of pseudo first-order and pseudo second-order have been studied for photocatalyst Mn_3_O_4_/ZnO/Eu_2_O_3_ nanoparticles. Studies revealed that the photocatalytic decomposition of the two studied dyes takes place via the pseudo first-order reaction, which is mentioned by Equation (2)

ln(C_i_/C_f_) = kt (2)

where C_f_ and C_i_ are the final concentration at time t and initial concentration, correspondingly, and k is the first order rate constant. MB degradation rate constants of 0.0042, 0.0215 and 0.0238 min^−1^ were achieved for the pH 4, pH 7 and pH 10, respectively (Figure 12a), whereas the MO degradation rate constants of 0.0023 min^−1^, 0.0081 min^−1^ and 0.01075 min^−1^ were obtained for pH = 4, 7 and 10, respectively (Figure 12b).

A comparison of superior photocatalytic removal efficacy of Mn_3_O_4_/ZnO/Eu_2_O_3_ photocatalyst for photodegradation of MB and MO dyes with already-reported photocatalytic systems containing ZnO nanoparticles, as tabulated in Table 1. Notably, the current Mn_3_O_4_/ZnO/Eu_2_O_3_ photocatalyst exhibited outstanding photodecomposition efficiency.

## 3. Experimental

### 3.1. Materials

With no further purification, raw materials of analytical grades such as (Zn(NO_3_)_2_·6H_2_O (Sigma-Aldrich, St. Louis, MO, USA), (Mn(CH_3_CO_2_)_2_·4H_2_O (Sigma-Aldrich, St. Louis, MO, USA) and (Eu(NO_3_)_3_·5H_2_O (Sigma-Aldrich, St. Louis, MO, USA) are employed. To make a model wastewater sample containing MB and MO (SD fine chemicals, India), dye solution is prepared with DW. Glasswares of BOROSIL make are utilized throughout this study. 

### 3.2. Preparation of Mn_3_O_4_/ZnO/Eu_2_O_3_ Photocatalyst

Stoichiometrically calculated amounts of Mn(CH_3_CO_2_)_2_·4H_2_O, Zn(NO_3_)_2_·6H_2_O and Eu(NO_3_)_3_·5H_2_O were dissolved in 10 mL of DW and 6 mL perished curd with vigorous stirring for 20 min. Thereafter, the resultant mixture is kept in a muffle furnace at 400 °C. After 10 min, a dark green powder is obtained and annealed at an identical temperature for 3 h.

### 3.3. Photocatalyst Characterization

The fabricated materials have been characterized by employing various common analyses, and all specifics about instruments were included in supporting data.

## 4. Conclusions

In summary, we have reported a facile fabrication of ternary heterojunction Mn_3_O_4_/ZnO/Eu_2_O_3_ nanocomposite via a facile one-pot combustion method. The characterization of the fabricated photocatalysts disclosed a crystalline nature and nano-size Mn_3_O_4_/ZnO/Eu_2_O_3_ nanocomposite. The fabricated sample has been examined as a photocatalyst for the photodegradation of MB and MO dyes and noxious industrial effluents. The Mn_3_O_4_/ZnO/Eu_2_O_3_ nanocomposite was tested for photodegradation of MB and MO dyes under natural solar irradiation; we have found that the photocatalyst is highly effective, and the photodegradation efficiency is affected by the changes in light source, catalyst amount, irradiation time, dye concentration and pH of the solution and the kinetics of the photocatalyst revealed 98% and 96% degradation of MB and MO dyes, respectively, in 150 min under the optimized reaction conditions. Therefore, further studies into the kinetics and fine-tuning of the economic and eco-friendly catalyst are in progress and shall be reported in the future. Lastly, the results disclosed that the Mn_3_O_4_/ZnO/Eu_2_O_3_ has been employed as an efficacious photocatalyst for the photodegradation of MB and MO under natural sunlight irradiation without any harmful impact on the environment.

## Figures and Tables

**Table 1:** Comparative studies of Mn_3_O_4_/ZnO/Eu_2_O_3_ for degradation of MB and MO dyes with several formerly reported analogous photocatalysts.

**Degradation of MB Dye**						
**Catalyst**	**Dye Concentration**	**Irradiation Source**	**Photocatalyst Dose**	**Time (min)**	**Degradation (%)**	**Ref.**
Mn_3_O_4_/ZnO/Eu_2_O_3_	5 ppm	Sunlight	15 mg	150	98	Herein
S-ZnO NPs	20 μM	Sunlight	30 mg	45	61.5	[42]
N/La-ZnO	15 ppm	Sunlight	50 mg	60	97	[43]
ZnO-SiO_2_	9 ppm	Sunlight	10 mg	90	97.8	[44]
ZnO NWs	10 ppm	Sunlight	100 mg/L	4320	100	[45]
Ce-Ag-ZnO/Fe_3_O_4_	10 ppm	UV lamp 15 W	30 mg	100	99	[46]
WO_3_/ZnO-rGO	5 ppm	Vis. 200 W	10 mg	90	94.1	[47]
Ag-ZnO/GO	15 ppm	Xe lamp 20 W	20 mg	180	85	[48]
TiO_2_/ZnO/rGO	0.3 ppm	Xe lamp 300 W	0.1 g/L	120	92	[49]
Mn-ZnO	10 ppm	UV Lamp	24 mg	90	60	[50]
1.5%Nd-Gd-ZnO	20 mg/L	Vis. 300 W	100 mg/L	120	93	[51]
rGO-ZnO	5 × 10^−4^ mol/L	Vis. light	100 mg/L	120	90	[52]
ZnO-CdO	3 × 10^−5^ mol/L	Xe lamp 250 W	-	360	97.8	[53]
ZnO NPs	15 ppm	Hg lamp 10 W	100 mg	120	90	[54]
Ag/ZnO	2 × 10^−5^ M	Xe lamp 100 W	100 mg	120	76	[55]
ZnO/AC	2 × 10^−5^ M	Hg lamp 30 W	25 mg	45	92.2	[56]
**Degradation of MO Dye**						
**Catalyst**	**Dye Concentration**	**Irradiation Source**	**Photocatalyst Dose**	**Time (min)**	**Degradation (%)**	**Ref.**
Mn_3_O_4_/ZnO/Eu_2_O_3_	5 ppm	Sunlight	15 mg	150	96	Herein
ZnO/biochar	25 ppm	UV Lamp 125 W	1 g/L	120	90.8	[57]
Gd-doped ZnO	16 ppm	UV Lamp 160 W	100 mg	90	85.3	[58]
ZnO-CuO	-	UV Lamp	30 mg	120	92.18	[59]
Ag-ZnO	10 ppm	Sunlight	120 mg	50	92.9	[60]
ZnO/NiAl-LDH	100 ppm	Hg lamp 100 W	100 mg	540	100	[61]
ZnONR-RGO	10 ppm	Hg lamp 250 W	45 mg	90	83	[62]
Ag-ZnONR	0.05 mM	UV Lamp 10 W	10 mg	120	88	[63]
ZnONR-Cu	0.03 mM	UV Lamp 10 W	5 mg	180	57.5	[64]
Mg-doped ZnO	20 ppm	Hg lamp 20 W	100 mg	120	73	[65]
Cu-doped ZnO	2 × 10^−6^ M	UV Lamp	10 mg	160	60	[66]
Fe_3_O_4_/ZnO-GO	1 × 10^−5^ M	Xe lamp 300 W	20 mg	150	92.8	[67]
ZSM-5/ZnO/Ag	5 ppm	UV Lamp 15 W	70 mg	180	90	[68]
Porous ZnO spheres	20 ppm	UV Lamp	0.2 g	120	96.3	[69]
Fe_3_O_4_/ZnO/Si_3_N_4_	50 ppm	Xe lamp	20 mg	90	96	[70]
Cu-ZnO NPs	20 ppm	UV Lamp 15 W	0.1 g	240	88	[71]

## Data Availability

Data are contained within the article.

## Supplementary Materials

The following are available online. Figure S1. UV–Vis absorption of the fabricated ZnO nanoparticles.

## Sample Availability

Samples of the compounds are not available from the authors.
