# Supplementary material for: Straightforward Synthesis of Mn3O4/ZnO/Eu2O3-Based Ternary Heterostructure Nano-Photocatalyst and Its Application for the Photodegradation of Methyl Orange and Methylene Blue Dyes
Source: Molecules. 2021 Jul 31;26(15):4661. doi: 10.3390/molecules26154661 (PMC8347762; doi:10.3390/molecules26154661)
Supplement: Supplementary file 1 [file molecules-26-04661-s001.zip › molecules-1229940-supplementary.pdf]

molecules

Supplementary Materials

Straightforward Synthesis of Mn_3O_4/ZnO/Eu_2O_3 Based Ternary Heterostructure Nano-photocatalyst and its Application for the Photodegradation of Methyl Orange and Methylene Blue Dyes

J. Pranesh Shubha[1,*], H. S. Savitha[2], Syed Farooq Adil[3,*], Mujeeb Khan[3], Mohammad Rafe Hatshan[3] , Kiran Kavalli[4] and Baji Shaik[5]

[1] Department of Chemistry, Don Bosco Institute of Technology, Mysore Road, Bangalore -560074, India.
[2] Department of Chemistry, SJB Institute of Technology, Kengeri, Bangalore -560060, India; hssavitha@sjbit.edu.in (H.S.S.)
[3] Department of Chemistry, College of Science, King Saud University, P.O. Box 2455, Riyadh 11451, Saudi Arabia; kmjeeb@ksu.edu.sa (M.K.); mhatshan@ksu.edu.sa (M.R.H.)
[4] Department of Mechanical and Automobile Engineering, School of Engineering and Technology, CHRIST Deemed to be University, Bangalore, India; kiran.k@christuniversity.in (K.K.)
[5] Department of Advanced Materials Engineering for Information & Electronics, Kyung Hee University, 1732 Deogyeong-daero, Giheung-gu, Yongin-si, Gyeonggi-do 446-701, Republic of Korea; shaikbaji2@khu.ac.kr (B.S)
* Correspondence: shubhapranesh@gmail.com (J.P.S.); sfadil@ksu.edu.sa (S.F.A.)

1.1. Characterization of photocatalysts

The as-synthesized heterostructures are characterized by UV–vis, FTIR, XRD, FESEM, and TEM. The XRD characterization is carried out using a Bruker diffractometer [Cu Kα (λ = 1.5406 Å) X-ray source]. The spectral characterization is carried out using a PerkinElmer UV–vis spectrometer and a Bruker IFS 66 v/S spectrometer for UV–vis and FTIR spectral analysis, respectively. The microscopic analysis such as SEM is carried out to understand the surface morphology, and particle size analysis is carried out by FESEM. TEM images are recorded with a transmission electron microscope, JEOL JEM2100 PLUS, operating at a 200 kV accelerating voltage.

1.2. Synthesis of Mn_3O_4

0.1M of $Mn(CH_3CO_2)_2.4H_2O$ was mixed thoroughly with 1.2 ml of curd for about 20 minutes later it was transferred to pre heated muffle furnace at 400 °C. The dark powder obtained was calcined at same temperature for about 3 hours.

1.3. Synthesis of ZnO

0.1M of $(Zn(NO_3)_2.6H_2O$ was mixed thoroughly with 0.8 ml of curd for about 20 minutes later it was transferred to pre heated muffle furnace at 400 °C. The pale yellow powder obtained was calcined at same temperature for about 3 hours.

1.4. Synthesis of Eu_2O_3

0.1M of $(Eu(NO_3)_3.5H_2O$ was mixed thoroughly with 0.8 ml of curd for about 20 minutes later it was transferred to pre heated muffle furnace at 400 °C. The dark powder obtained was calcined at same temperature for about 3 hours.

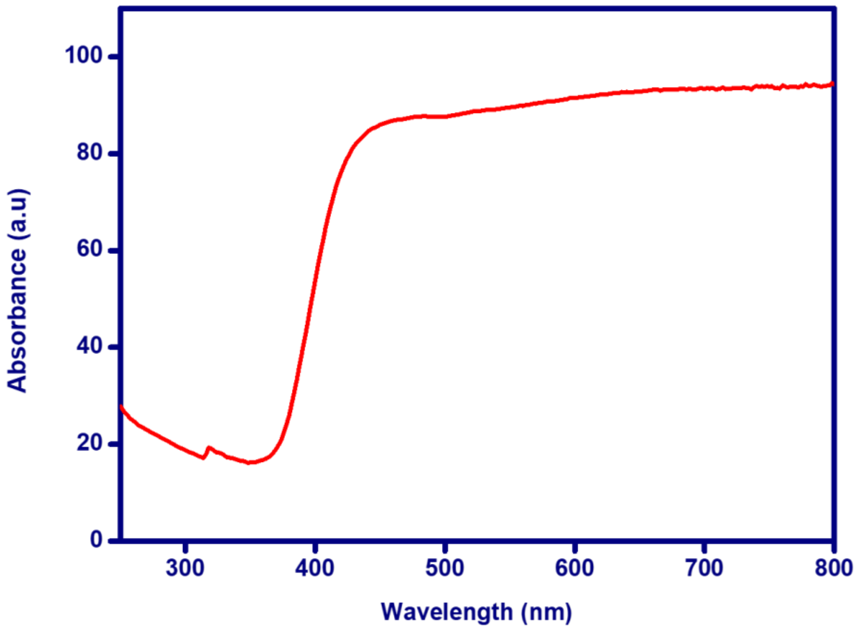

Fig. S1. UV–Vis absorption of the fabricated ZnO nanoparticles.
